# Enhanced brain parcellation via abnormality inpainting for neuroimage-based consciousness evaluation of hydrocephalus patients by lumbar drainage

**DOI:** 10.1186/s40708-022-00181-5

**Published:** 2023-01-19

**Authors:** Di Zang, Xiangyu Zhao, Yuanfang Qiao, Jiayu Huo, Xuehai Wu, Zhe Wang, Zeyu Xu, Ruizhe Zheng, Zengxin Qi, Ying Mao, Lichi Zhang

**Affiliations:** 1 Department of Neurosurgery, Huashan Hospital, Shanghai Medical College, Fudan University, Shanghai, 200040 China; 2 National Center for Neurological Disorders, Shanghai, 200040 China; 3 Shanghai Key Laboratory of Brain Function and Restoration and Neural Regeneration, Shanghai, 200040 China; 4 Neurosurgical Institute of Fudan University, Shanghai, 200040 China; 5 Shanghai Clinical Medical Center of Neurosurgery, Shanghai, 200040 China; 6 State Key Laboratory of Medical Neurobiology and MOE Frontiers Center for Brain Science, School of Basic Medical Sciences and Institutes of Brain Science, Fudan University, Shanghai, 200040 China; 7 School of Biomedical Engineering, Shanghai Jiao Tong University, Shanghai, 200030 China

**Keywords:** Structural imaging, Brain parcellation, Hydrocephalus, Traumatic brain injury, Consciousness

## Abstract

Brain network analysis based on structural and functional magnetic resonance imaging (MRI) is considered as an effective method for consciousness evaluation of hydrocephalus patients, which can also be applied to facilitate the ameliorative effect of lumbar cerebrospinal fluid drainage (LCFD). Automatic brain parcellation is a prerequisite for brain network construction. However, hydrocephalus images usually have large deformations and lesion erosions, which becomes challenging for ensuring effective brain parcellation works. In this paper, we develop a novel and robust method for segmenting brain regions of hydrocephalus images. Our main contribution is to design an innovative inpainting method that can amend the large deformations and lesion erosions in hydrocephalus images, and synthesize the normal brain version without injury. The synthesized images can effectively support brain parcellation tasks and lay the foundation for the subsequent brain network construction work. Specifically, the novelty of the inpainting method is that it can utilize the symmetric properties of the brain structure to ensure the quality of the synthesized results. Experiments show that the proposed brain abnormality inpainting method can effectively aid the brain network construction, and improve the CRS-R score estimation which represents the patient’s consciousness states. Furthermore, the brain network analysis based on our enhanced brain parcellation method has demonstrated potential imaging biomarkers for better interpreting and understanding the recovery of consciousness in patients with secondary hydrocephalus.

**Supplementary Information:**

The online version contains supplementary material available at 10.1186/s40708-022-00181-5.

## Introduction

Hydrocephalus refers to an excessive accumulation of cerebrospinal fluid (CSF) in the ventricle system and/or the subarachnoid space, which is a common complication of traumatic brain injuries. When hydrocephalus is critically aggravated, the structure of cortical cells may be destroyed progressively [1], affecting the level of consciousness and even leading to disorders of consciousness (DOC). Cerebrospinal fluid (CSF) shunt operations are the most used method to treat hydrocephalus. Shunt procedures can address pressure on the brain caused by hydrocephalus and relieve some symptoms, but fail to completely restore neurological dysfunction in some cases even if deformations of ventricle can be restored. In clinical practice, lumbar cerebrospinal fluid drainage (LCFD) [2–5] is recommended to apply for hydrocephalus patients with the consciousness evaluation using JFK Coma Recovery Scale-Revised (CRS-R), which can be used to determine if placing a shunt can benefit on recovery of consciousness. Based on the CRS-R, DOC patients can be further diagnosed into different states of consciousness ranging from unresponsive wakefulness syndrome (UWS) (also named vegetative state, VS) to minimally conscious state (MCS) and emergence from minimally conscious state (EMCS). Despite its wide acceptance, the application of LCFD has bottleneck in applications as conventional CRS-R assessment is highly subjective, recent studies reported that 37–43% of patients were misdiagnosed due to the use of untrained physicians and spontaneous fluctuation within patients [6].

Due to the above-mentioned limitations, new consciousness evaluation approaches are critical for the clinical treatment of patients with hydrocephalus. Magnetic resonance imaging (MRI) has emerged as the primary noninvasive and effective technique to observe in vivo neural structures and functions. Many attempts for measuring hydrocephalus patients’ consciousness using different modalities of MRIs have been developed to facilitate clinical treatment [7–10]. For example, functional connectivity (FC) in the default mode network (DMN) is significantly correlated with the prognosis of consciousness [7]. Moreover, in a diffusion tensor imaging (DTI) study, patients with NPH exhibited significantly different fractional anisotropy (FA) values in the corpus callosum and corticospinal tract compared with healthy controls [9]. In previous studies, we developed a novel machine-learning-based method to track the changes in CRS-R scores based on multimodal (T1 and DTI) images before and after LCFD. Based on the collected T1 images, there were 5 single-region of interest (ROI) features that correlated with CRS-R scores. The thalamus region was one of the 5 ROIs. In addition, there are 3 scalar features related to the IC based on the DTI images; these features have a significant correlation with the patient’s change in consciousness level. This suggests the importance of the IC in assessing CRS-R scores and evaluating the effect of LCFD. With the proposed model, we can predict the CRS-R scores for an unseen patient before and after LCFD [11].

In this study, we further investigate brain network analysis using resting-state fMRI (rs-fMRI) images of hydrocephalus patients, and conducted a comprehensive study of the brain network in correlation with consciousness status. Generally, brain network analysis based on rs-fMRI is implemented through the following major steps: 1. brain parcellation based on the provided atlas template; 2. blood oxygen level-dependent (BOLD) signal extraction based on the parcellation results to segment all the necessary brain regions in the rs-fMRI; 3. extraction of FC information based on the Pearson's correlation computation of BOLD signal information between each brain region; and 4. construction of the brain network. Therefore, brain parcellation of hydrocephalus patients plays an important role in whole-brain network analysis, which lays the foundation for subsequent processes.

Given that it is generally impractical to prepare for manual brain parcellation, the development of an accurate and automatic segmentation method that can perform in an unsupervised manner is critical. Normally, this automated technique is implemented using the single-atlas registration method due to its simplicity; specifically, the label map of the target brain image is estimated by spatially aligning the atlas template using a certain image registration method [12]. Considerable effort has been devoted to this field, and many of these efforts focus on developing and incorporating registration methods, such as Demons [13], HAMMER [14], FNIRT [15], LDDMM [16], SyN [17], etc. Recently, deep-learning-based methods, such as VoxelMorph [18] have also been developed; these methods can obtain more accurate registration results with much faster computation times. In addition, Wang et al. proposed LT-Net [19] which combines segmentation and registration tasks to further improve parcellation performance.

**Fig. 1:**
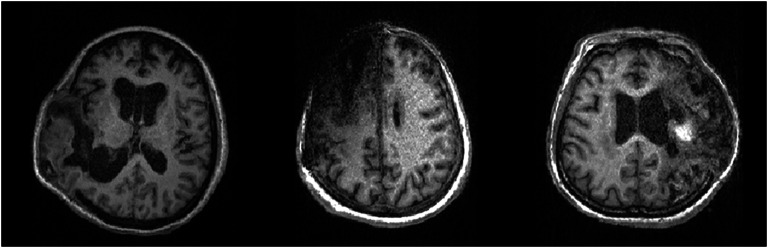
Exemplary T1 images of the brains of post-traumatic hydrocephalus patients with brain lesions and large deformations

However, due to the large deformations and lesion erosions in the brain images of post-traumatic hydrocephalus patients (shown in Fig. 1), it is much more challenging to perform brain parcellation tasks for these patients than the normal patients when directly using the single-atlas registration method. In addition, few studies of brain parcellation for images of hydrocephalus patients can be found in the literature. Pertinently, Ledig et al. [20] proposed a fully automatic segmentation method based on expectation–maximization. This form of segmentation outperformed the state-of-the-art methods on normal subjects, but the authors reported failed cases when parcellating the images of hydrocephalus patients. In addition, Ren et al. [21] combined registration and segmentation based on deep learning as a two-stage parcellation framework, and Qiao et al. [22] further proposed an end-to-end framework for further improving parcellation performance. However, their works were not focused on whole-brain parcellation and were implemented in a supervised manner, which required manually annotated brain images to construct the segmentation models and is generally impractical to prepare.

In this study, we intended to develop an unsupervised brain parcellation method, which can achieve whole-brain segmentation of hydrocephalus images and aid the subsequent brain network analysis. To resolve the above-mentioned challenges, we introduced a novel brain abnormality inpainting method that can restore the deformed or lesion-erosion parts to normal, and constructed an inpainted normal brain corresponding to its hydrocephalic version. In this way, the brain parcellation process can be implemented by first registering the atlas template to the inpainted images, and then normalizing it to the original input images. The intermediate step using the inpainted images can greatly reduce registration difficulty and therefore lead to improvements in parcellation tasks. Recent developments in image inpainting were generally based on convolutional neural networks [23] and generative adversarial networks (GANs) [24] The aim was to restore the damaged image parts while ensuring that the synthesized images have perceptual similarity to the original images. For example, Pathak et al. [25] proposed a context encoder that adopts a CNN and adversarial learning to achieve image inpainting, and Yang et al. [26] further extended their work by incorporating neural patches [27] to synthesize high-resolution results. Liu et al. [28] also improved convolution in the image inpainting process and proposed a partial convolution operation that can conduct inpainting for irregular missing parts. In this paper, we further investigated the properties of the images of hydrocephalus patients and designed our brain abnormality inpainting method to ensure the robustness of the inpainting performance. To our knowledge, this is the first work in the literature to conduct inpainting on images of such complicated abnormal parts of the brain that suffer from both lesion erosion and large deformations.

The main contributions of our work are as follows: (1) we developed a novel abnormality inpainting method that can generate synthesized and inpainted images of hydrocephalus patients; these images can be utilized for brain parcellation and lay the foundations for subsequent brain network analysis. (2) Using the obtained rs-fMRI images, we performed a comprehensive study of the constructed FC network with each patient’s CRS-R scores using the small-world network analysis and a least absolute shrinkage and selection operator (LASSO) regression method. We also conducted experiments to demonstrate the validity of our proposed inpainting method, its effectiveness in brain network construction, and its efficacy in the evaluation of consciousness in clinical practice.

## Materials and methods

### Participants

This study included 28 patients with secondary mild hydrocephalus treated at Huashan Hospital, Fudan University from January 2013 to March 2018. The patients included 20 men and 8 women (age range, 18–78 years; average age, 47.44 years). The demographic information and the clinical characteristics are shown in Table 1. All patients underwent MRI scans before LCFD, and follow-up scans were performed after 3 days of LCFD. The concurrent CRS-R scores were evaluated by a trained neurosurgeon before the MRI scan was performed to determine the level of consciousness. Clinicians classified patients with improved CRS-R scores (several times independent assessments from 6 neurosurgeons) after LCFD into the "favorable" group, whereas the remainder of the patients were classified into the "unfavorable" group. The patients were further diagnosed into different states of consciousness based on the CRS-R scale: UWS present moments of arousal, during which they open their eyes and produce complex behavior reflexes without any signs of intentional behavior; MCS- was defined by the presence of low-level behavioral responses (i.e., visual pursuit, localization of noxious stimulation or other contingent behavior such as emotional smiling or crying); MCS+ was defined as the presence of command following, intelligible verbalization or any yes/no responses; EMCS was defined as patients who recover the ability of functionally communication. (Additional file 1: Table S1) Informed consent was obtained from all patients for the use of their information, medical records, and MRI data.

**Table 1:** Demographic and clinical information of all the recruited patients with secondary mild hydrocephalus

Category	Favorable	Unfavorable	*p* value
Age (year): mean ± std	47.53 ± 13.48	45.09 ± 14.61	0.6547
Gender: male/female	14/3	7/4	0.2639
CRS-R scores before LCFD: mean ± std	11.71 ± 6.00	16.27 ± 7.99	0.0960
CRS-R scores after LCFD: mean ± std	15.82 ± 5.03	16.27 ± 7.99	0.8560

The patients were classified into two categories: “favorable” indicates patients whose CRS-R scores increased after LCFD and whose consciousness levels improved after LCFD, and “unfavorable” indicates patients whose CRS-R scores did not improve after LCFD. For more information about the CRS-R scores and the diagnosis of the states of consciousness, see Additional file 1: Table S1

### Image acquisition and preprocessing

All MRI data were acquired on a 3 T SIEMENS MR scanner at Huashan Hospital, Fudan University. Two structural modalities, i.e., T1 and rs-fMRI, were used in this research. For T1 images, the scanning parameters were slices = 176, slice thickness = 1 mm, no interslice gap, repetition time (TR) = 2300 ms, echo time (TE) = 2.98 ms, inversion time = 900 ms, noninterpolated voxel size = 1 × 1 × 1 mm^3^, flip angle (θ) = 9°, and field of view (FOV) = 240 × 256 mm^2^. For rs-fMRI, TR/TE/θ = 2000 ms/35 ms/90°, FOV = 256 × 256 mm^2^, matrix size = 64 × 64, slices = 33 with 4 mm thickness, gap = 0 mm, and scans = 200.

The collected T1 and rs-fMRI images were preprocessed as follows. For T1 images, histogram equalization and N4 Bias correction were implemented using the FMRIB Software Library (FSL) toolkit [29] to ensure image quality. For fMRI images, we used DPARSFA [30] software in MATLAB 9.2 for preprocessing. The first 10 time points for each subject were discarded to ensure the stability of the collected data. Other preprocessing tasks were completed as follows: time correction, head movement correction (translation on *X*-, *Y*-, *Z*-axes < 2 mm; rotation < 2°), spatial standardization, and spatial smoothing using a Gaussian kernel (full width at half maximum = 8 mm). We also obtained the mean echo-planar imaging (EPI) data from the preprocessed rs-fMRI data and estimated the transformation matrix to align the T1 image to the mean EPI data using FLIRT in FSL, which is used in the brain parcellation procedure described in Sect. 2.3.1.

Note that apart from the collected hydrocephalus brain image dataset mentioned above, we also used other images as auxiliary data for aiding the registration and image inpainting tasks. For example, we adopt the Alzheimer’s Disease Neuroimaging Initiative (ADNI) dataset [31], which contains normal MRI brain images without hydrocephalus symptoms. The aim was to train the inpainting model, which is further introduced in Sect. 2.3.1. Specifically, we randomly selected 20 images from the ADNI dataset for our study and employed the same preprocessing procedure as that used for the hydrocephalus dataset, such as histogram equalization and N4 Bias correction. The images were then cut into 2D slices, resulting in 3500 slices for training. In addition, we used the Automated Anatomical Labeling (AAL) [32] atlas template, including the T1 template image and 90 brain regions excluding the cerebellum. The template image was utilized in the process described in Sect. 2.3.1 for our brain atlas to achieve brain parcellation and was preprocessed by histogram matching to ensure parcellation performance.

### Methods

In this section, we introduce our framework of FC network analysis based on hydrocephalus brain images, which includes two main steps: image processing for network construction and network analysis with correlations to consciousness status. As previously mentioned in Sect. 1, brain network construction requires a brain parcellation process, of which the overall pipeline is illustrated in Sect. 2.3.1. The details of the abnormality inpainting method are introduced in Sect. 2.3.2. After image parcellation and brain network construction were performed based on the patients’ fMRI data, we further investigated the clinical assessments of hydrocephalus patients based on the obtained FC information. In Sect. 2.3.3, we present the small-world network analysis, which represents the global characteristics of the brain connectivity network. In Sect. 2.3.4, we focus on the individual FC information for the estimation of consciousness level.

#### Hydrocephalus brain parcellation

**Fig. 2:**
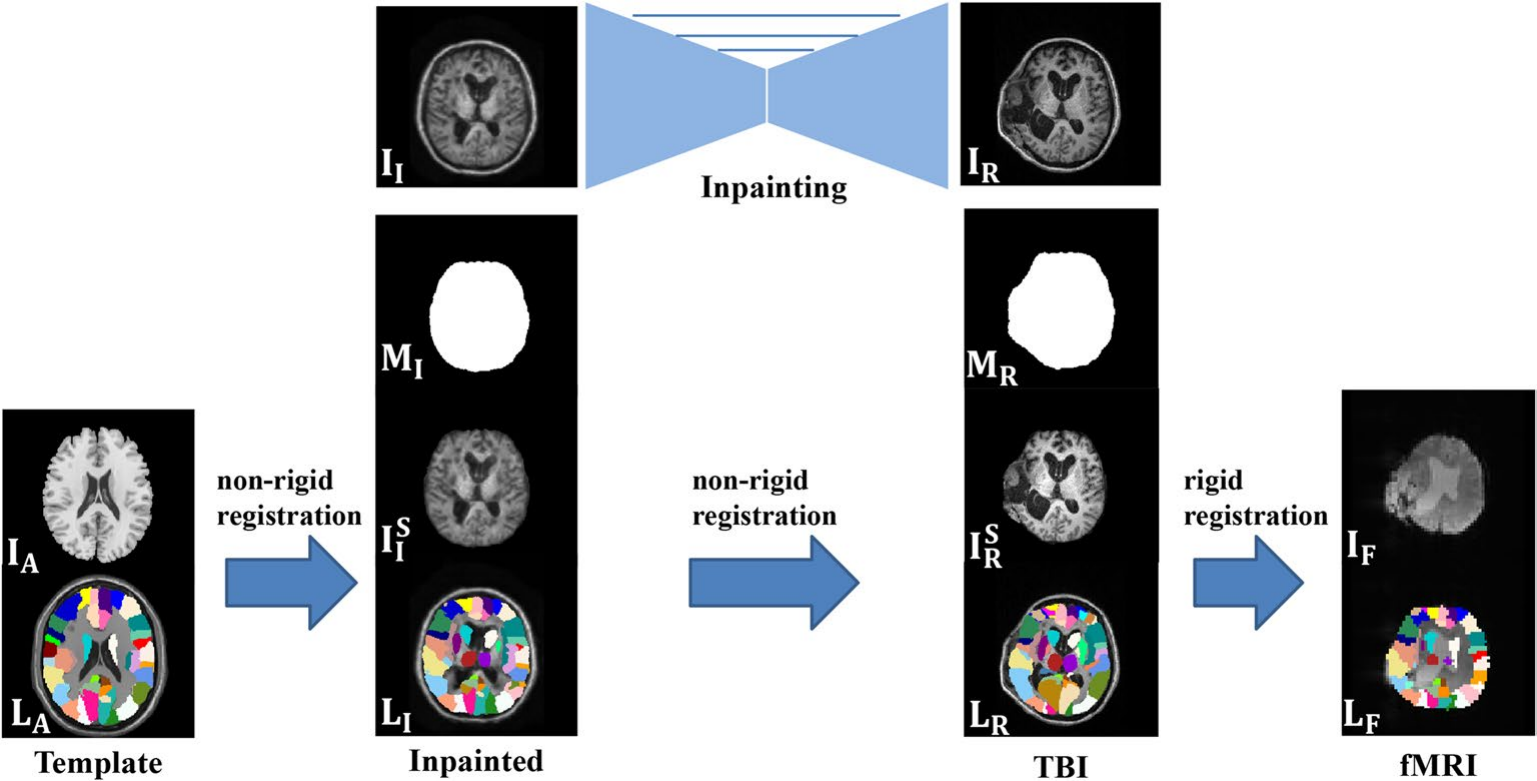
Hydrocephalus brain parcellation framework with the aid of the abnormality inpainting method. The three major registration steps are designed to warp the label map from the atlas template (left) to the mean EPI of the fMRI image (right)

The overall pipeline of the hydrocephalus brain parcellation is presented in Fig. 2, and our main goal was to parcellate fMRI images based on the provided atlas template to obtain their corresponding label maps. Generally, image parcellation consists of several major registration steps. First, we performed abnormality inpainting and skull stripping to obtain the brain image of the original *T*1 data and the inpainted results. The details are further presented in Sect. 2.3.2. Here, the original *T*1 image is designated ${I}_{\mathrm{T}}$, and the inpainted normal image is designated ${I}_{\mathrm{N}}$. Note that before the brain image was fed into the abnormality inpainting network, we manually erased the irregular structure of the brain where large deformation and lesion regions occur, which was restored by the inpainting process. Given that ${I}_{\mathrm{N}}$ indicates the restored anatomical structure of a normal brain image, we can apply the classical skull-stripping tool BET in the FSL toolkit [29] to generate its brain mask ${M}_{\mathrm{N}}$. Next, we conducted nonrigid registration from ${I}_{\mathrm{N}}$ to ${I}_{T}$ and obtained a deformation field ${\phi }_{{I}_{\mathrm{N}}\to {I}_{T}}$. Then, we warped ${M}_{\mathrm{N}}$ using the deformation field ${\phi }_{{I}_{\mathrm{N}}\to {I}_{T}}$ to obtain ${\mathrm{M}}_{T}$, which is the brain mask of ${I}_{T}$. We used the mask ${M}_{T}$ to strip the skull and obtain the stripped hydrocephalus brain image ${I}_{T}^{\mathrm{S}}$. The nonrigid registration and warping processes were implemented using the Advanced Normalization Tools (ANTs) toolkit [33].

Next, we used the AAL template [32] ${I}_{\mathrm{A}}$ with 90 brain regions ${L}_{\mathrm{A}}$, which is introduced in Sect. 2.2 as our brain atlas. We performed a total of two nonrigid registrations and one rigid registration, as shown in Fig. 2, which were also achieved using the ANTs toolkit. Then, we performed nonrigid registration from ${I}_{\mathrm{A}}$ to ${I}_{\mathrm{N}}^{\mathrm{S}}$ and another nonrigid registration from ${I}_{\mathrm{N}}^{\mathrm{S}}$ to ${I}_{T}^{\mathrm{S}}$ to obtain the deformation fields ${\phi }_{{I}_{\mathrm{A}}\to {I}_{\mathrm{N}}^{\mathrm{S}}}$ and ${\phi }_{{I}_{\mathrm{N}}^{\mathrm{S}}\to {I}_{T}^{\mathrm{S}}}$. By superimposing the above two deformation fields, we can obtain the deformation field from ${I}_{\mathrm{A}}$ to ${I}_{T}^{\mathrm{S}}$ denoted as ${\phi }_{{I}_{\mathrm{A}}\to {\mathrm{I}}_{T}^{\mathrm{S}}}$. We also estimated the segmentation result ${L}_{T}$ of the input *T*1 image by warping ${L}_{\mathrm{A}}$ using ${\phi }_{{I}_{\mathrm{A}}\to {I}_{T}^{\mathrm{S}}}$. Then, we performed rigid registration to obtain the transformation matrix ${\phi }_{{I}_{T}^{\mathrm{S}}\to {I}_{\mathrm{F}}}$ from the input *T*1 brain image to the corresponding fMRI image. We finally warped ${L}_{T}$ using the transformation matrix to estimate the label map of the fMRI image ${L}_{\mathrm{F}}$. In this way, we can construct the brain FC network for the subsequent analysis based on the obtained ${L}_{\mathrm{F}}$.

#### Brain abnormality inpainting for images from patients with hydrocephalus

**Fig. 3:**
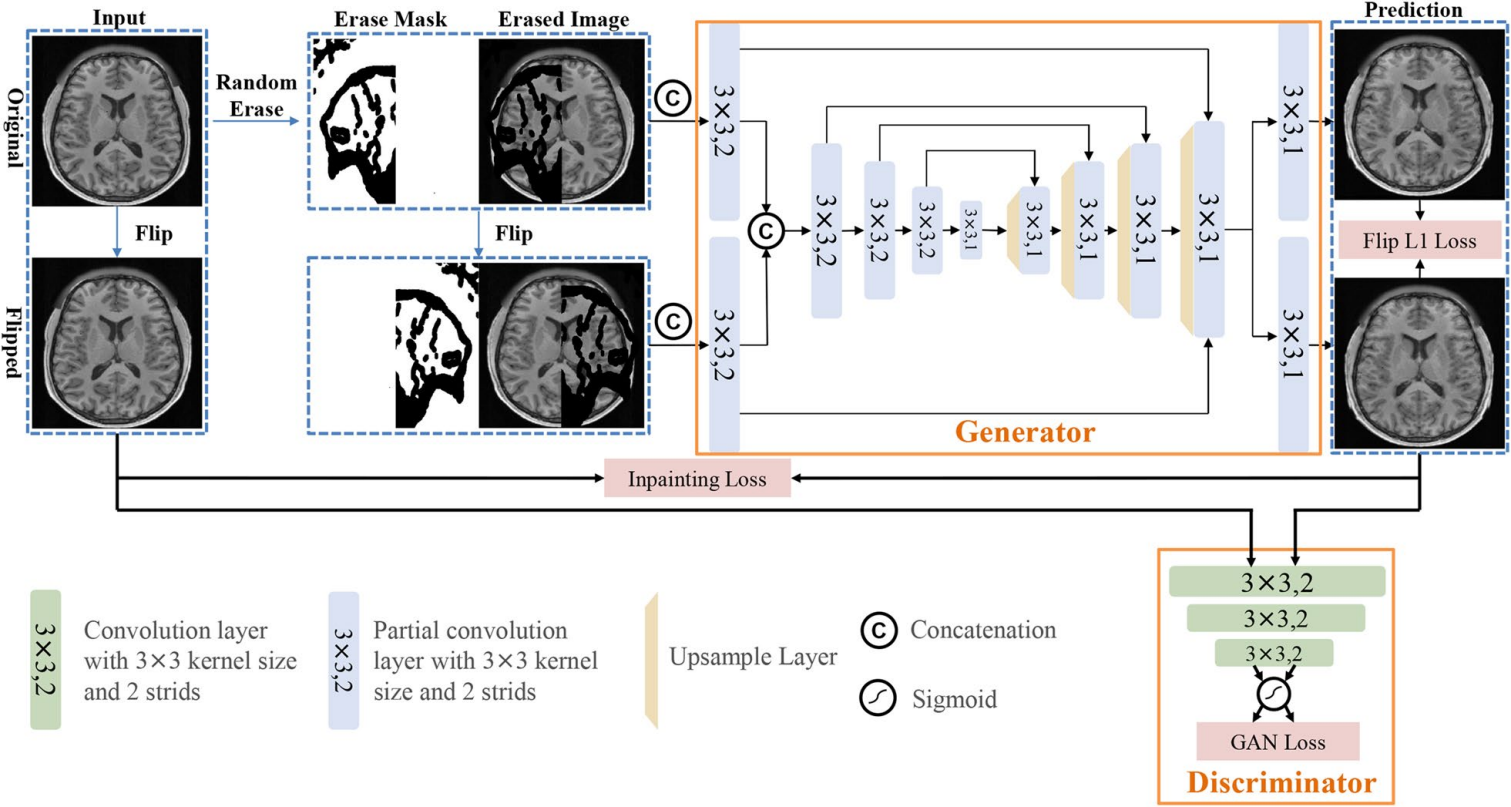
The overall pipeline of the brain abnormality inpainting method for images from hydrocephalus patients. The framework consists of a UNet-like generator and a discriminator. The input of UNet is the concatenation of the random erased image with its erase mask and the flipped version

The hydrocephalus inpainting method described here was developed based on the GAN framework, which consists of a generator and a discriminator. The generator was used to produce the inpainted version of the input images, while the discriminator was employed to ensure the validity of the inpainted results and provide feedback to the generator to further improve inpainting performance. The framework is shown in Fig. 3, where the UNet-like structure was used as the generator and the encoder in the lower right corner was used as the discriminator.

To ensure the effectiveness of the abnormality inpainting process, we utilized the symmetric properties in the anatomical structure of the human brain and designed a novel inpainting framework with the corresponding data augmentation strategy for training data preparation. Normally, the training data for abnormality inpainting are collected by randomly generating erase masks that are applied to the intensity image. Then, the erase mask with the erased image is concatenated as a two-channel pair to be fed into the network for training. Here, we imposed the constraint that the normal images are generally symmetric in anatomical structure, and the input of our abnormality inpainting network is the original concatenation of the erased image with its flipped version. Using this method, it was easier for the network to learn the brain structure of the erased part based on the input of the flipped version and the similarity of the two outputs from the network.

Next, the two sources were concatenated after a partial convolution layer and fed into the inpainting network, which was designed as an end-to-end UNet framework, including encoders and decoders, where all convolution layers are replaced by partial convolution. Compared with the convolution layer, the input of the partial convolution layer consists of the erased image and corresponding binary mask; additionally, the calculation process includes a partial convolution operation and mask updating step, which demonstrated effectiveness in natural image restoration [28]. In the partial convolution operation, the input image first undergoes elementwise multiplication with the corresponding binary mask before convolution. In the mask updating step, the pixel value is set as 1 if the output of the partial convolution operation has values in that location. By continuously stacking the partial convolution layers, the input erase mask eventually becomes 1, and the erased part in the image can also be filled. After the operations of the encoder and decoder of the UNet-like generator, the image with the erased part is inpainted.

Once the inpainting network outputs the inpainted result and its flipped version, they are then concatenated as the input of the discriminator. After the encoder of multiple convolution layers, the outputs of the discriminator represent the possibility that the discriminator interprets the inputs to be true images. Similarly, the raw image is also used as input to the discriminator to train the recognition ability of the discriminator.

The output of the network is constrained by a series of loss functions that contain pixel-level accuracy (ACC) and context-level smoothness to improve the reliability of the inpainting process. The loss functions used in the training stage are noted as follows:

1 $$\begin{array}{c}{L}_{\mathrm{inpainting}}=\,{L}_{\mathrm{valid}}+{6L}_{\mathrm{erase}}+0.05{L}_{\mathrm{perceptual}}+120\left({L}_{{\mathrm{style}}_{\mathrm{out}}}+{L}_{{\mathrm{style}}_{\mathrm{comp}}}\right)+0.1{L}_{\mathrm{tv}},\end{array}$$

where the first two items are *L*1 loss in the network output for the erased pixels and non-erased pixels and the input pixel in the erased part is zero; therefore, the coefficient of ${L}_{\mathrm{erase}}$ is greater than ${L}_{\mathrm{valid}}$. The perceptual loss ${L}_{\mathrm{perceptual}}$ computes the *L*1 distance between the high-level features of output and ground truth using the VGG-16 [34] model (pretrained using ImageNet dataset [35], which is written as follows:

2 $$\begin{array}{c}{L}_{\mathrm{perceptual}}=\frac{1}{{\mathrm{N}}_{{I}_{\mathrm{out}}}}{\Vert {V(I}_{\mathrm{out}})-{V(I}_{\mathrm{gt}})\Vert }_{1},\end{array}$$

where $V$ represents the feature extractor of the pretrained VGG-16 model. To ensure the consistency of styles between the output and the ground truth to make the inpainted image more realistic, the style-loss term performs an autocorrelation on the high-level feature as follows:

3 $$\begin{array}{c}{L}_{\mathrm{style}}=\frac{1}{{C}^{2}}{\Vert {{{V(I}_{\mathrm{out}})}^{T}V(I}_{\mathrm{out}})-{{{V(I}_{\mathrm{gt}})}^{T}V(I}_{\mathrm{gt}})\Vert }_{1},\end{array}$$

where $C$ represents the channel number of ${V(I}_{\mathrm{out}})$. The last loss item is the total variation (TV) loss ${L}_{\mathrm{tv}}$, which is the smoothing penalty for the output image.

To ensure the robustness of the brain abnormality inpainting process, we adopted some supervision priors to guide the training of the inpainting model. Specifically, we used *L*1 loss to constrain the gap between the result of the raw image ${I}_{\mathrm{out}}$ and the result of the flipped image ${I}_{\mathrm{outflip}}$:

4 $$\begin{array}{c}{L}_{\mathrm{flip}}=\frac{1}{{\mathrm{N}}_{{I}_{\mathrm{out}}}}{\Vert {I}_{\mathrm{out}}-{I}_{\mathrm{outflip}}\Vert }_{1}.\end{array}$$

In this way, the total loss for inpainting includes the inpainting loss of raw image ${\mathrm{L}}_{\mathrm{inpainting}}$, the inpainting loss of flipped image ${L}_{\mathrm{inpainting}}^{\mathrm{F}}$ and the *L*1 loss of flipping ${L}_{\mathrm{flip}}$:

5 $$\begin{array}{c}{L}_{\mathrm{total}}={L}_{\mathrm{inpainting}}+{L}_{\mathrm{inpainting}}^{\mathrm{F}}+{L}_{\mathrm{flip}}. \end{array}$$

Once the inpainted images were produced using the generator, we also designed a corresponding discriminator in an encoder manner for generative adversarial training. The discriminator’s input is the generative image from the inpainting model and the ground truth, whereas the output is the probability that represents the input image as the ground truth. In this way, the binary cross-entropy loss function is used to train the discriminator:

6 $$\begin{array}{c}{L}_{\mathrm{D}}=-\left(\mathrm{log}\left(1-\mathrm{D}\left(\left[{I}_{\mathrm{out}},{I}_{\mathrm{outflip}}\right]\right)\right)+\mathrm{log}\left(\mathrm{D}\left(\left[{I}_{\mathrm{gt}},{I}_{\mathrm{gtflip}}\right]\right)\right)\right).\end{array}$$

Given that the purpose of the generator is to produce synthesized images that make the discriminator difficult to distinguish from real images, we added another loss function for the generator, defined as:

7 $$\begin{array}{c}{L}_{\mathrm{G}}=-\mathrm{log}\left(\mathrm{D}\left(\left[{I}_{\mathrm{out}},{I}_{\mathrm{outflip}}\right]\right)\right).\end{array}$$

Note that we used ADNI datasets [31] as our training datasets, which were first preprocessed to normalize the image domains of ADNI data to the target hydrocephalus datasets, preventing the potential domain shift issues in the inpainting process. Details of the preprocessing work are presented in Sect. 3.1. During the training stage, we alternately trained the generator and the discriminator, where the loss function of the generator is ${L}_{\mathrm{total}}+0.1{L}_{\mathrm{G}}$ and the loss function of the discriminator is ${L}_{\mathrm{D}}$. In the end, a balance was reached between the discriminator and the generator, and the generator can produce high-quality inpainting results.

#### Small-world network analysis

After implementing brain parcellation and obtaining the label map of the fMRI image, we can extract node time courses of fMRI signals within the ROIs from the preprocessed fMRI image. Pearson’s correlation coefficients between every connected node were estimated to construct the FC network, which is presented as a symmetric matrix with a size of 90 × 90. Then, we applied the threshold to the correlation matrix and eliminated the weak correlation to obtain the binary adjacency matrix $\mathrm{A}$, where ${\mathrm{A}}_{\mathrm{i},\mathrm{j}}$ is 1 when the correlation of node i and node j is greater than the threshold. In our study, we chose 10 different thresholds to apply to the connectivity matrix and obtained 10 different binary adjacency matrices for each sample.

Small-world networks have a shorter characteristic path length than regular networks but greater local interconnectivity than random networks. Some studies have shown a correlation between small-world characteristics of the brain based on brain networks and consciousness [36, 37], especially in DOC patients [38]. Two characteristics are primarily used to measure the network: the characteristic path length, $L,$ and the clustering coefficient, $C$. $L$ is the average path length of all node pairs in the network, and $\mathrm{C}$ is the mean value of the aggregation coefficient of all nodes, where the aggregation coefficient of a node is the score value obtained by dividing the actual number of edges by the maximum possible number of edges. These network characteristics are further normalized by comparing their counterpart of the random network and can be defined using the following equation:

8 $$\begin{array}{c}\lambda =\frac{L}{{L}_{\mathrm{random}}},\gamma =\frac{C}{{C}_{\mathrm{random}}},\sigma =\frac{\gamma }{\lambda },\end{array}$$

where ${L}_{\mathrm{random}}$ and ${C}_{\mathrm{random}}$ are the averages of $L$ and $C$ calculated from 10 different random networks. Given that random networks have short $L$ and low $C$ values, and small-world networks have short $L$ and high $C$ values, we can obtain an approximate range of the above three characteristics: *λ* ≈ 1, *γ* > 1, and $\sigma$>1 [39].

To compare the difference in the small-world network before and after LCFD, we calculated the small-world characteristics for each patient at two time points. The small-world analysis was performed before and after LCFD for patients in both favorable and unfavorable groups. For the adjacency matrix under 10 different thresholds, we performed 10 rounds of significant difference analysis and compared the difference between favorable and unfavorable groups. All the above operations were implemented using GRETNA V2.0 [40].

#### LASSO for CRS-R score regression

Some studies have shown that certain correlations exist between brain FCs and the patient’s consciousness status [36, 37]. Here we further explore the relationship between FCs and the CRS-R scores by applying a LASSO regression for feature selection and CRS-R score regression based on FCs, for the expectation of using the brain FCs to objectively evaluate the level of consciousness in patients with DOC. The LASSO method implement regression based on the following equation:

9 $$\begin{array}{c}\underset{w}{\mathrm{min}} J\left(w\right)={\Vert y-Xw\Vert }_{2}^{2}+k{\Vert w\Vert }_{1},\end{array}$$

where matrix $X$ represents the feature set from all patients, the size of one patient is 90 $\times$ 89/2 = 4005 according to the region numbers of the AAL atlas template, $y$ is the column vector of the CRS-R scores, and $w$ is the estimated efficient vector. *L*-1 regularizer is added to enforce sparse learning of the features, which can be used for selecting the most significant features for the CRS-R score.

### Evaluation methodologies

In this paper, we intend to evaluate the effectiveness of our proposed consciousness evaluation method for hydrocephalus patients, which includes the abnormal brain inpainting method and the brain network analysis. For the inpainting method, we follow the widely recognized evaluation protocols on natural images, and calculate the peak signal–noise ratio (PSNR), structural similarity (SSIM), *L*1 and *L*2 errors between the real ADNI brain images and the inpainted ADNI brain images. Corresponding visualized results between the baseline (without the flipping-guidance strategy) and the proposed inpainting methods are also presented for further demonstrating the improvements of the proposed inpainting method.

Evaluation of brain parcellation and brain network analysis is slightly more complicated. As previously stated in the Introduction section, given that manual brain parcellation of hydrocephalus brain images is generally impractical, the evaluation of our segmentation results cannot be implemented by simply comparing them with the provided ground truth. Therefore, we aimed to demonstrate whether better brain parcellation with abnormal inpainting can facilitate subsequent brain network analysis and result in better consciousness assessments than those without abnormal inpainting.

We used nested cross-validation to conduct regression and categorization experiments. Specifically, we established two nested loops in the leave-one-out cross-validation settings. First, each patient was selected as the test set and used to verify the ACC of the regression model, and the other patients were used as the training set. Second, in the training stage, each patient was chosen in turn to validate the performance of the regression model. The parameters were determined by all validation cases using grid searching. After the parameters were determined, they were adopted for regression on all training datasets. After training, the test set was predicted by the trained model, and the predicted results before and after LCFD were compared with the ground truth. Note that in the regression experiments, we used mean square errors (MSEs) to evaluate the ACC of the regression model according to the CRS-R scores.

## Results

Our experimental results for the evaluation of are presented in threefold: first, we demonstrated the validity of our novel brain abnormality inpainting method for patients with brain lesions and the application of this method in the brain parcellation task. The inpainted normal images were visualized to demonstrate the effectiveness of the inpainting works. Second, we constructed the FC network based on the results of brain parcellation and further conducted brain network analysis to investigate small-world characteristics. We also applied the LASSO model to regress the CRS-R score based on the FC information from the neuroimaging data and show that our abnormality inpainting method can significantly improve the quality of brain parcellation, which greatly helps the performance of subsequent processes, such as CRS-R score estimation. We also used the LASSO method to identify the most discriminative features that are highly correlated with the consciousness level in hydrocephalus patients.

### Brain parcellation

**Fig. 4:**
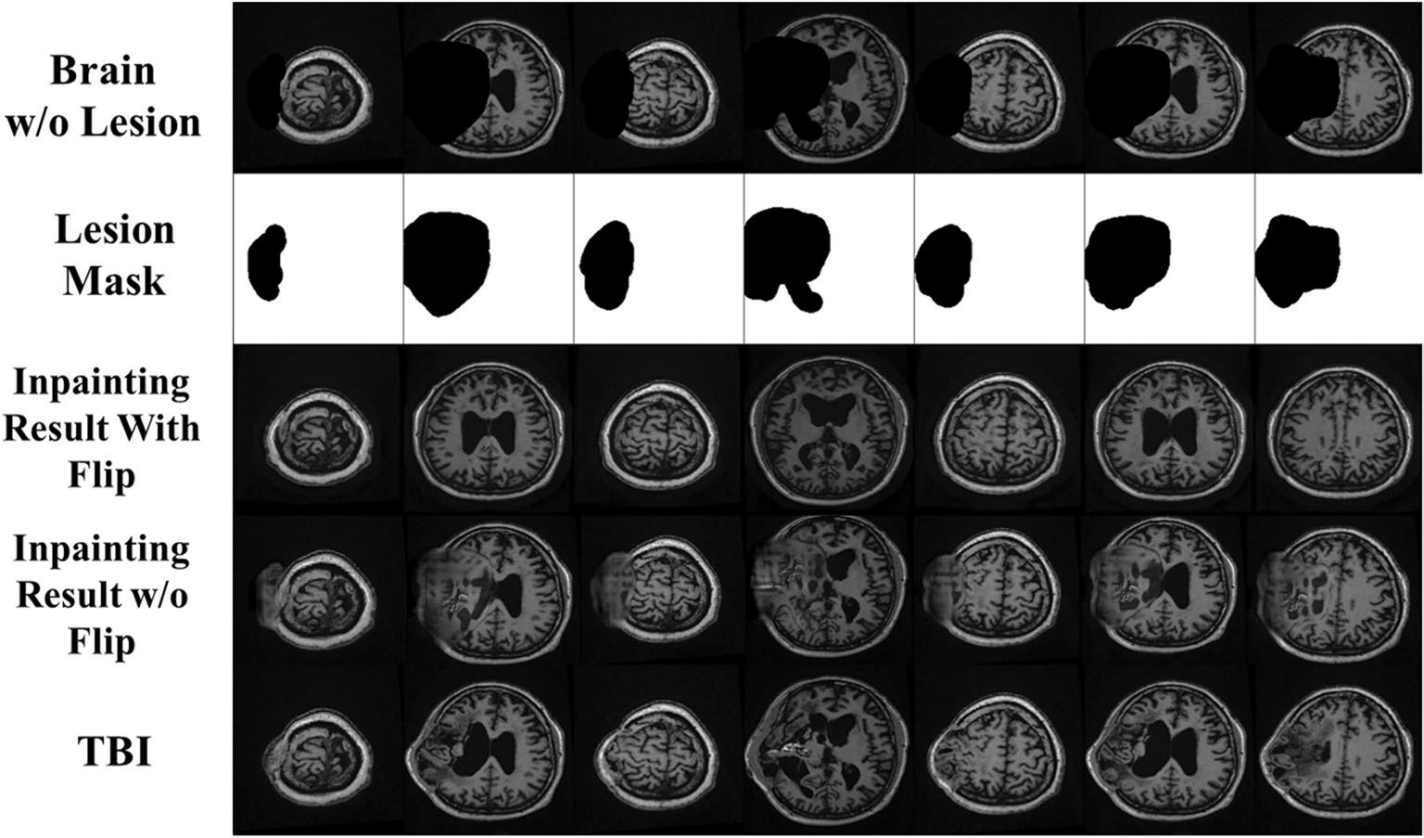
Exemplary brain inpainting results for the images from hydrocephalus patients. Row 1 shows the input images, and row 2 shows the input image without the lesion region (erase mask) presented in row 3. Rows 4 and 5 show the output of the inpainting network without and with the flipping priors

Here, we evaluated the performance of our constructed inpainting model, which was applied to the collected images of hydrocephalus patients and compared with the conventional inpainting method without the input of flipping. The quantitative and qualitative inpainting results comparison are presented in Table 2 and Fig. 4, respectively. In Table 2, the first row shows the results of the baseline inpainting method, and the second row shows the results of the proposed inpainting method. The experimental results reveal that the inpainting quality has been improved by a large margin due to the utilization of the symmetry of human brains. Compared with the baseline method, the proposed inpainting method brings much performance gain in terms of all the evaluation metrics.

**Table 2:** Quantitative comparison between the baseline inpainting method without flipping and the proposed inpainting method

Method	PSNR	SSIM	L1 error	L2 error
Inpainting without flipping	21.42	0.8756	0.0971	0.0466
Proposed inpainting with flipping	25.02	0.9355	0.0611	0.0103

In Fig. 4, we visualize several inpainting results on patients with hydrocephalus: the first row shows the original MRI scan, the second row shows the image with the lesion region erased, and the lesion mask is presented in row 3. The last two rows show the inpainting results excluding and including the prior guidance from the flipped images. Moreover, simply using the conventional inpainting method cannot guarantee the quality of the reconstruction work, as the inpainted areas consist of only blurred and meaningless artifacts. On the other hand, our novel abnormality inpainting method utilized the priors from the flipped images, which can effectively restore the erased part of the image and produce realistic normal brain images instead.

**Fig. 5:**
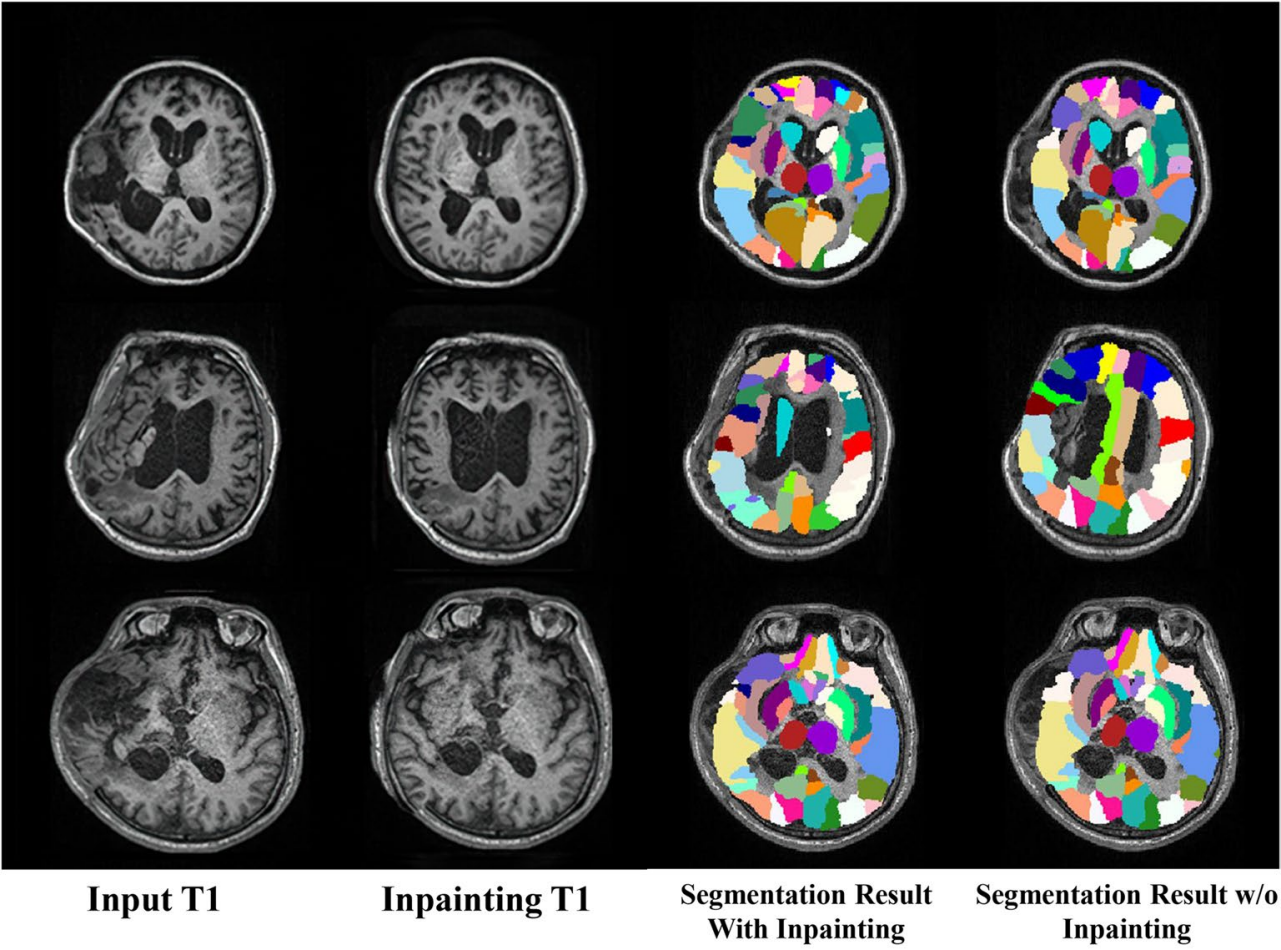
Segmentation result. From left to right are the input T1 images, inpainted results, and segmentation results with and without inpainting

After obtaining the inpainted brain image, we followed the pipeline shown in Fig. 2 and used the AAL template as our atlas to segment brain regions. The results are presented in Fig. 5. Note that all registration operations were implemented using ANTs [33]. In Fig. 5, the first column is the input raw image, the second column is the inpainting result and the third column is the segmentation result by inpainting. To further verify the effectiveness of our method, we also conducted brain parcellation without including the inpainting process, in which we directly register from the template to the input T1 image and warp the label map using the registration field. The corresponding result is shown in the fourth column of Fig. 5. Significant differences in the brain parcellation results are noted between the third and fourth columns. In addition, the parcellation results in the fourth column are not aligned with the actual anatomical structure of the images, indicating that it is almost impossible to establish direct nonrigid registration from the template to the input hydrocephalus T1 images due to the large deformations and lesions. On the other hand, when using the inpainted normal images as an intermediate role for indirect registration, alignment can generally be implemented.

### Small-world network analysis

**Fig. 6:**
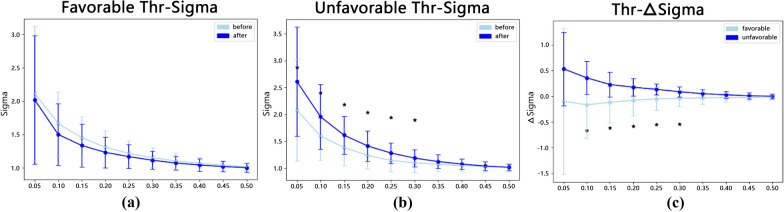
Small-world results. **a** and **b** are the σ distributions of patients in the favorable and unfavorable groups, respectively. **c** is the ∆σ distribution

Here, we constructed FC networks based on the brain parcellation results and further conducted the small-world analysis to explore whether the differences in FC networks correlate with the improvements in CRS-R scores after LCFD surgery. The dataset was divided into two groups (favorable and unfavorable), and each group had the small-world characteristics $\sigma$ under different thresholds before and after LCFD. The results are shown in Fig. 6a (favorable) and Fig. 6b (unfavorable). Note that ‘*’ denotes a significant difference in *p-*value (less than 0.05) between the two different groups of time points. We calculated the average value of $\sigma$ under different thresholds for each sample, and the distribution of the average value for each group is shown in Table 3.

**Table 3:** Distribution of average σ values

	Before LCFD	After LCFD	*p* value
Favorable	1.3147 ± 0.2506	1.2515 ± 0.2463	0.4461
Unfavorable	1.2721 ± 0.2628	1.4339 ± 0.2856	0.0090

To compare the difference between patients in the favorable group and those in the unfavorable group, we calculate $\Delta \sigma = {\sigma }_{\text{after}}-{\sigma }_{\text{before}}$, which represents the difference in small-worldness before and after LCFD. The result is shown in Fig. 6c. The average value under different thresholds was also calculated, and the distribution is shown in Table 4.

**Table 4:** Distribution of average ∆σ values

	Favorable	Unfavorable	*p-*value
∆σ	-0.0632 ± 0.3235	0.1618 ± 0.1584	0.04869

### LASSO regression analysis

In this section, we train a LASSO regression model and obtain the parameters that minimize the loss function in Eq. 9. To evaluate the performance of the regression, we computed the Pearson’s correlation coefficient (CC) between the predicted and real CRS-R scores. Also, we computed the mean square errors (MSEs) between our CRS-R predictions and the real ones, and *R*^2^ score of the regression to quantify the degree of linear correlation between CRS-R predictions and the real ones.

**Fig. 7:**
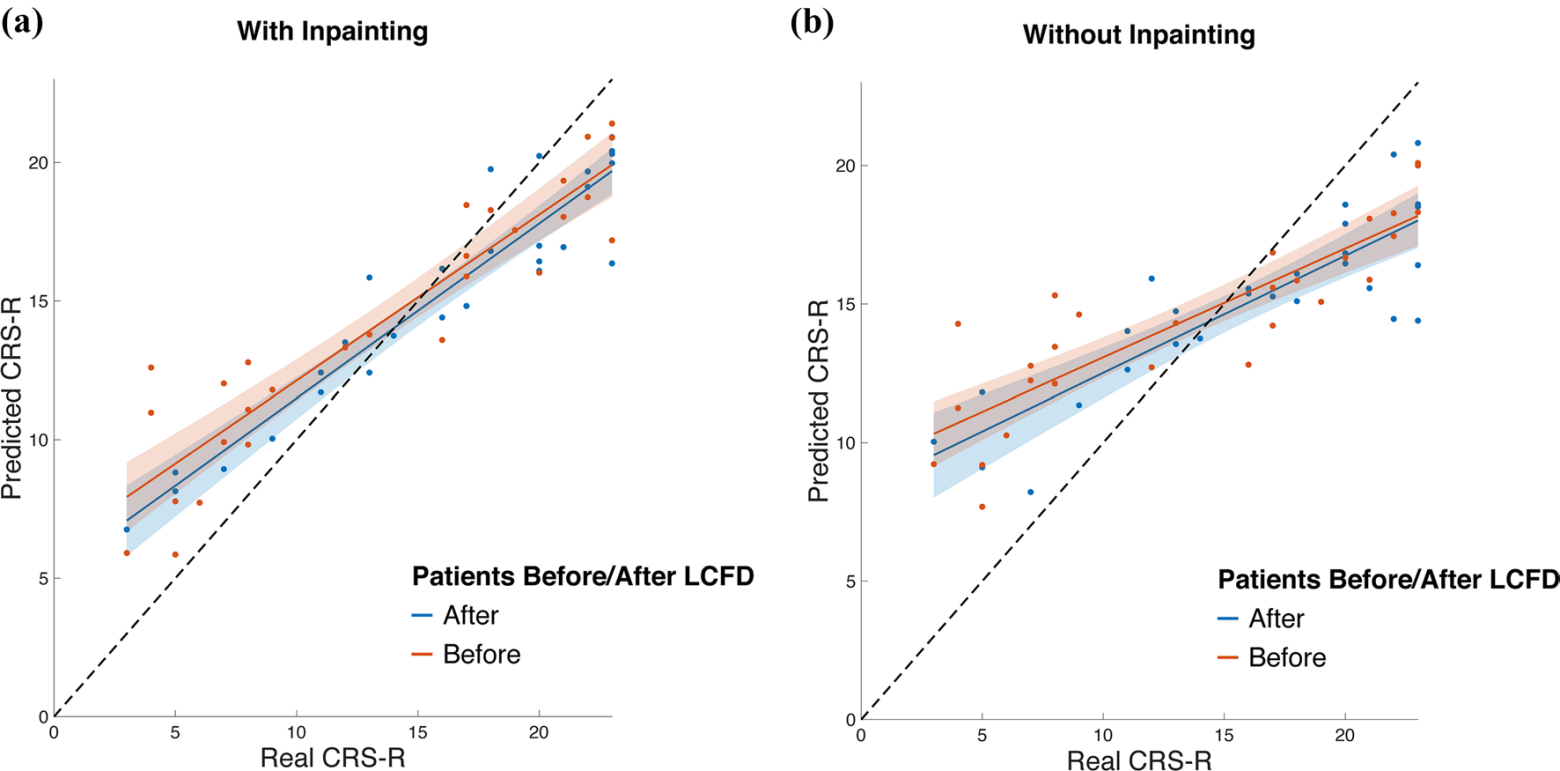
Regression results using the LASSO method. **a** Segmentation is implemented using the inpainting method. **b** The inpainting method is not used during segmentation

To further validate the effectiveness of our brain parcellation process based on the proposed abnormality inpainting method, we compared the regression result using the label map from our method and the direct registration method without inpainting, which is shown in Table 5. The regression result is also depicted in the scatter plots in Fig. 7. The figure also demonstrates that the features obtained from the abnormality inpainting method can achieve better regression accuracy, which indicates the effectiveness of the abnormality inpainting model. The improvements can also be concluded from Table 5, where we computed Pearson’s correlation coefficient (CC) and MSEs between the predicted and the real CRS-R scores. It is shown that the method using the inpainting approach greatly outperformed the one without inpainting.

**Table 5:** Comparison of methods with and without inpainting in regressing CRS-R scores

	CC	MSE	R^2^
w/o inpainting	0.8549	18.5792	0.5812
w inpainting	0.9333	9.3029	0.7903

**Fig. 8:**
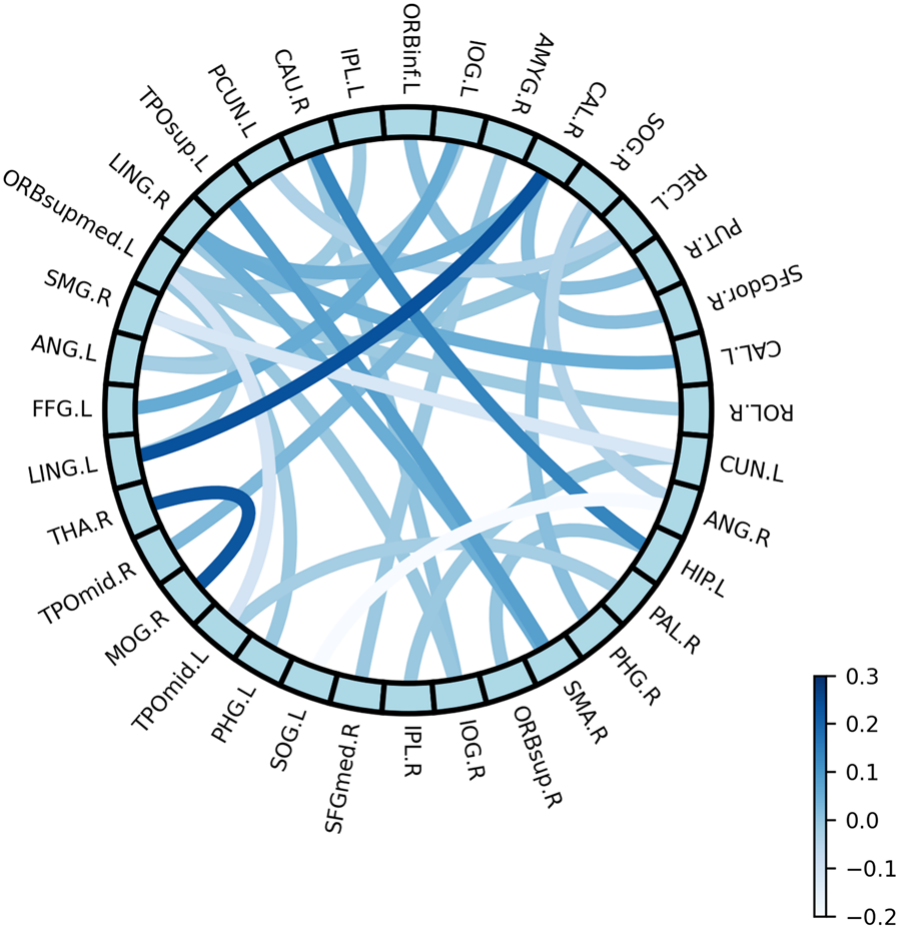
Selected features based on $w$

In the regression model, vector $w$ represents the importance of the corresponding feature for CRS-R score regression. After the feature selection and regression, many elements in $w$ were near zero, indicating that the feature is negligible for regression. Here, we set the threshold as $t=|\overline{w }|$. The features with absolute value coefficients $|w|$ greater than the threshold were selected as important for CRS-R score regression. In our study, the features used in regression were all forms of FC information between two brain regions, and 32 features were selected by $w$. The most discriminative features were illustrated in Fig. 8, where the color of the connecting line between 2 regions represents the value of $|w|$. Additional details of the selected FC information are summarized in Table 6.

**Table 6:** The list of the most discriminative features from LASSO, as well as their correlation with the CRS-R scores (^*^p < 0.05, ^**^p < 0.01)

Region 1	Region 2	*w*	CC	*p*-value
Frontal_mid_orb_l	Rolandic_Oper_R	− 0.011	− 0.278	0.038^*^
Hippocampus_L	Frontal_Sup_Orb_R	− 0.001	− 0.146	0.283
Parahippocampal_l	Frontal_mid_orb_l	− 0.008	− 0.256	0.057
Amygdala_R	Frontal_Sup_Medial_R	− 0.009	− 0.225	0.096
Calcarine_r	Frontal_Sup_R	0.013	0.424	0.001^**^
Lingual_L	Frontal_mid_orb_l	− 0.010	− 0.166	0.221
Lingual_L	Calcarine_r	0.237	0.463	0.001^**^
Lingual_R	Supp_motor_Area_R	0.051	0.438	0.001^**^
Lingual_R	Frontal_mid_orb_l	− 0.009	− 0.117	0.390
Lingual_R	Calcarine_L	0.052	0.355	0.007^**^
Lingual_R	Calcarine_r	0.046	0.444	0.001^**^
Occipital_Sup_R	ParaHippocampal_R	0.015	0.310	0.020^*^
Occipital_Inf_l	Frontal_mid_orb_l	− 0.017	− 0.197	0.145
Occipital_Inf_R	Frontal_mid_orb_l	− 0.007	− 0.134	0.325
Fusiform_L	Occipital_Inf_l	0.057	0.378	0.004^**^
Parietal_Inf_l	Frontal_mid_orb_l	− 0.012	− 0.102	0.452
Parietal_Inf_r	Cuneus_L	− 0.007	− 0.392	0.003^**^
SupraMarginal_R	Cuneus_L	− 0.120	− 0.320	0.016^*^
Angular_L	Frontal_mid_orb_l	− 0.007	− 0.140	0.303
Angular_L	Rectus_L	− 0.004	− 0.291	0.029^*^
Angular_L	SupraMarginal_R	− 0.023	− 0.240	0.074
Angular_R	Occipital_Sup_L	− 0.257	− 0.348	0.009^**^
Angular_R	Occipital_Sup_R	− 0.044	− 0.327	0.014^*^
Precuneus_L	Rectus_L	− 0.037	− 0.333	0.012^*^
Caudate_R	Hippocampus_L	0.137	0.270	0.044^*^
Caudate_R	Occipital_Inf_R	0.004	0.260	0.053
Putamen_r	Frontal_Inf_orb_l	0.014	0.248	0.066
Thalamus_R	Occipital_mid_R	0.227	0.475	0.001^**^
Temporal_pole_sup_L	Supp_motor_Area_R	0.081	0.429	0.001^**^
Temporal_pole_Mid_L	Frontal_mid_orb_l	− 0.111	− 0.204	0.131
Temporal_pole_Mid_L	Pallidum_r	− 0.023	− 0.233	0.083
Temporal_pole_Mid_R	Calcarine_r	0.028	0.402	0.002^**^

## Discussion

### Abnormality inpainting for hydrocephalus brain parcellation

Brain network analysis based on rs-fMRI data requires the prerequisite works of brain parcellation, which aims to locate and segment all the required brain regions in the fMRI images so that the BOLD signals corresponding to these regions can be extracted. However, manual annotations of these brain regions are quite labor-intensive and impractical; therefore, an automatic brain parcellation method is in high demand. Due to the high complexity of anatomical structures in hydrocephalus brain images, it is challenging to achieve segmentation tasks, especially for regions influenced by large abnormal deformities and lesion erosions.

In this paper, we proposed to develop a novel abnormality inpainting method for aiding the brain image parcellation of hydrocephalus. The main idea was to generate an inpainted normal version of the input brain image of hydrocephalus, which can be used to reduce the difficulty in registering the given atlas template to the target, making it feasible for brain parcellation and FC network construction. We conducted experiments to show the effectiveness of the inpainting method in Sect. 3.1, and we also demonstrated that the inpainting method can greatly aid the estimation performance in subsequent processes, especially for brain parcellation and consciousness evaluations.

### FC network with human consciousness

Based on the results of brain parcellation, brain network analysis for brain images of hydrocephalus was conducted to investigate small-world characteristics. The small-world characteristics of the favorable and unfavorable groups are significantly different. Thus, we can use small-world network analysis to predict the effectiveness of VP shunts in patients with hydrocephalus. LCFD, as a traditional method of determining the effectiveness of VP shunt surgery, is invasive, and the results are only available after 3 to 7 days. Thus, the ideal time for disease diagnosis and treatment might be missed. A noninvasive, fast MR image-based small-world network analysis approach has the potential to change the treatment selection guidelines for patients with hydrocephalus.

In previous studies, we proposed a novel machine learning method to find the most discriminative features from T1 and DTI neuroimages. Then, we built the regression model to regress CRS-R scores to quantify human consciousness [11]. Although DTI can capture the influence of hydrocephalus in white matter regions, it fails to quantify such variations in gray matter regions. Therefore, here, we included the rs-fMRI data as the additional modality, which was designed to detect FC among different gray matter regions [41] and provide a more thorough investigation of hydrocephalus symptoms in the whole brain.

Through the small-world network method, modularity at the global level was found to be reduced in hydrocephalus patients compared to that in healthy controls, suggesting a disturbance in the optimal balance between segregation and integration networks. Clinically, proper treatment of hydrocephalus, such as LCFD, can effectively restore cognitive dysfunction and enhance the level of consciousness. However, how does the organization of large-scale functional networks change during the treatment of hydrocephalus and the recovery of consciousness? Accordingly, we applied graph theory to investigate changes in the topological properties of whole-brain functional networks. Based on the results, we found a different trend of small-world topology between favorable and unfavorable outcomes during treatment. Patients in both groups showed classical small-worldness (σ > 1) at baseline. However, those in the favorable group exhibited decreased small-worldness at follow-up, whereas those in the unfavorable group exhibited increased small-worldness at follow-up. These differences in small-worldness resulted from the decrease in whole-brain integration and a reduced probability of high-degree nodes. Our findings are consistent with previous research on propofol-induced loss of consciousness [36]. Propofol can disrupt brain FC in both cortical and subcortical regions; therefore, the increase in small-worldness σ may be related to the random rewiring of a more locally connected graph under pharmacological disruption and can sequentially cause more local and less globally integrated information processing. Our findings indicated that surgical interventions can reverse the decrease in global integration and may suggest the restoration of brain organization. Moreover, network properties were altered in several regions that are associated with conscious processing (particularly in the medial parietal and frontal regions, as well as in the thalamus) [42]. Through small-world networks, Northoff et al. [43] also demonstrated temporal and spatial differences in brain network structure between conscious and unconscious people. While some may argue that the functional connectivity is derived from the BOLD signal which reflects hemodynamic events other than directly reflects the neural activity like other electrophysiological assessments does, recent studies found strong correlation between EEG spectral measures and functional connectivity measures derived from resting-state fMRI, supporting the brain network analysis in DOC patients[53].

The sparse learning method has demonstrated a strong capability to select features to track CRS-R scores. Compared with independent feature selection (e.g., principal component analysis (PCA)), LASSO has improved regression performance significantly by integrating feature selection and regression into a unified framework. As shown in Table 6, we computed the correlation between each selected feature and the CRS-R score for all patients.

We identified 1 ROI feature of the thalamus that was correlated with CRS-R scores. The thalamocortical system has long been crucial to human consciousness [44–48]. In those studies, for example, altered FA values between hydrocephalus patients and normal controls were found in the thalamus before and after VP shunt surgery. Our findings provide more evidence that highlights the structural changes in the thalamus in the clinical assessment of consciousness. We also found the connections between visual perception and motor regions correlate with the level of consciousness by identifying the importance of the lingual gyrus and supplementary motor area. This supports the theory that the higher-order sensorimotor circuit of the brain network is the infrastructure for consciousness [49].

Moreover, the temporal–parietal joint region (TPJ) and precuneus have been frequently discussed in the literature related to consciousness. Our previous work demonstrated that the strength of FC between the posterior medial cortex (PMC, including posterior cingulate cortex and precuneus) and TPJ varied significantly between DOC patients and normal controls, and the strength of cross-functional connectivity between the hemispheres of the PCC-TPJ was further found to be significantly correlated with the level of consciousness [50]. The strength of FC between PMC and the left lateral parietal cortex, which is part of the TPJ, was significantly associated with patient outcomes [7] 7. In our work, 7 scalar features were selected and then related to both TPJ and precuneus (including the supramarginal gyrus and angular gyrus), which suggests the importance of the TPJ in assessing CRS-R scores and evaluating the effect of LCFD.

### Limitations and potential future research

Admittedly, some factors were present that limited our work, and these limitations can be addressed in future work. First, the current work was based on a relatively small number of subjects. Although we have demonstrated our work in the current collected hydrocephalus dataset, more subjects need to be recruited for further validation. Second, the current inpainting method is based on 2D slices of MRI scans. The development of a 3D abnormality inpainting method can produce better-synthesized results; however, more training data and computational resources would be required to achieve this task. Third, no external validation of the level of consciousness from other modalities, especially the evidence from electrophysiological tests such as EEG, SEP or ABR. The correlation between our fMRI results and electrophysiological assessments of consciousness should be investigated in our future research.

## Conclusions

In this paper, we developed a novel abnormality inpainting method that can generate synthesized and inpainted images for hydrocephalus patients. To our knowledge, we are among the first to conduct brain FC network analysis for hydrocephalus brain images. Specifically, we can conduct small-world network analysis and LASSO to perform brain FC network analysis and consciousness assessment; moreover, we can identify optimal FC features that are highly correlated to predicting CRS-R scores. The proposed brain network construction and analysis solution for consciousness assessment of hydrocephalus patients can achieve promising estimations and has high potential in clinical practice. In future studies, we will continue developing brain segmentation methods (e.g., [22, 52]) for hydrocephalus brain images and focus on utilizing the brain functional network to further explore the applications of consciousness assessments in clinical studies.

## Supplementary Information

**Additional file 1.** Clinical information of 28 patients with secondary mild hydrocephalus.

## Data Availability

Data are not available due to ethical restrictions. Due to the nature of this research, participants of this study did not agree for their data to be shared publicly, so supporting data is not. The code that support the findings of this study are available from the corresponding authors, Zengxin Qi and Lichi Zhang, upon reasonable request.

## References

[1] Governale LS (2008). Techniques and complications of external lumbar drainage for normal pressure hydrocephalus. Neurosurgery.

[2] Zahl SM (2011). Benign external hydrocephalus: a review, with emphasis on management. Neurosurg Rev.

[3] Del BM (2010). Neuropathology and structural changes in hydrocephalus. Dev Disabil Res Rev.

[4] Daou B (2016). Revisiting secondary normal pressure hydrocephalus: does it exist? A review. Neurosurg Focus.

[5] Marmarou A (2005). Diagnosis and management of idiopathic normal-pressure hydrocephalus: a prospective study in 151 patients. J Neurosurg.

[6] Giacino JT, Kalmar K, Whyte J (2004). The JFK Coma Recovery Scale-Revised: measurement characteristics and diagnostic utility. Arch Phys Med Rehabil.

[7] Qin P (2015). How are different neural networks related to consciousness?. Ann Neurol.

[8] Huang Z (2014). The self and its resting state in consciousness: an investigation of the vegetative state. Hum Brain Mapp.

[9] Osuka S (2010). Evaluation of ventriculomegaly using diffusion tensor imaging: correlations with chronic hydrocephalus and atrophy. J Neurosurg.

[10] Khoo HM (2016). Default mode network connectivity in patients with idiopathic normal pressure hydrocephalus. J Neurosurg.

[11] Huo J (2020). Neuroimage-based consciousness evaluation of patients with secondary doubtful hydrocephalus before and after lumbar drainage. Neurosci Bull.

[12] Chao-Gan Y, Yu-Feng Z (2010). DPARSF: a MATLAB toolbox for "Pipeline" data analysis of resting-state fMRI. Front Syst Neurosci.

[13] Thirion JP (1998). Image matching as a diffusion process: an analogy with Maxwell’s demons. Med Image Anal.

[14] Shen D, Davatzikos C (2002). HAMMER: hierarchical attribute matching mechanism for elastic registration. IEEE Trans Med Imaging.

[15] Andersson J, Smith S, Jenkinson M (2008) FNIRT-FMRIB’s non-linear image registration tool. Human Brain Mapping

[16] Beg MF et al (2005) Computing large deformation metric mappings via geodesic flows of diffeomorphisms. Int J Computer Vis 61(2):139–157

[17] Avants BB (2008). Symmetric diffeomorphic image registration with cross-correlation: evaluating automated labeling of elderly and neurodegenerative brain. Med Image Anal.

[18] Balakrishnan G (2019). VoxelMorph: a learning framework for deformable medical image registration. IEEE Trans Med Imaging.

[19] SW et al (2020) LT-Net: Label Transfer by Learning Reversible Voxel-Wise Correspondence for One-Shot Medical Image Segmentation. In: IEEE/CVF Conference on Computer Vision and Pattern Recognition (CVPR), p 9159–9168

[20] Ledig C (2015). Robust whole-brain segmentation: application to traumatic brain injury. Med Image Anal.

[21] XR et al (2020) Robust Brain Magnetic Resonance Image Segmentation for Hydrocephalus Patients: Hard and Soft Attention. In: IEEE 17th International Symposium on Biomedical Imaging (ISBI)

[22] Qiao Y (2021). Robust Hydrocephalus Brain Segmentation via Globally and Locally Spatial Guidance.

[23] Krizhevsky A et al (2017) Imagenet classification with deep convolutional neural networks. Communications of the ACM 60(6):84–90

[24] Goodfellow IJ et al (2014) Generative adversarial nets (Advances in neural information processing systems, Red Hook, NY Curran, pp 2672–2680

[25] DP et al (2016) Context Encoders: Feature Learning by Inpainting. In: IEEE Conference on Computer Vision and Pattern Recognition (CVPR)

[26] CY et al (2017) High-Resolution Image Inpainting Using Multi-scale Neural Patch Synthesis. In: IEEE Conference on Computer Vision and Pattern Recognition (CVPR)

[27] Li C, Wand M (2016) Combining Markov Random Fields and Convolutional Neural Networks for Image Synthesis. In: IEEE Conference on Computer Vision and Pattern Recognition (CVPR)

[28] Liu G et al (2018) Image inpainting for irregular holes using partial convolutions. In: Proceedings of the European Conference on Computer Vision (ECCV)

[29] Smith SM (2004). Advances in functional and structural MR image analysis and implementation as FSL. Neuroimage.

[30] Wei W (2016). More severe extratemporal damages in mesial temporal lobe epilepsy with hippocampal sclerosis than that with other lesions: a multimodality MRI study. Medicine.

[31] Jack CJ (2008). The Alzheimer’s disease neuroimaging initiative (ADNI): MRI methods. J Magn Reson Imaging.

[32] Tzourio-Mazoyer N (2002). Automated anatomical labeling of activations in SPM using a macroscopic anatomical parcellation of the MNI MRI single-subject brain. Neuroimage.

[33] Avants BB (2011). A reproducible evaluation of ANTs similarity metric performance in brain image registration. Neuroimage.

[34] Simonyan K, Zisserman A (2014). Very Deep Convolutional Networks for Large-Scale Image Recognition. Comp Sci.

[35] Russakovsky O (2015). ImageNet large scale visual recognition challenge. Int J Comp Vision.

[36] Schroter MS (2012). Spatiotemporal reconfiguration of large-scale brain functional networks during propofol-induced loss of consciousness. J Neurosci.

[37] Uehara T (2014). Efficiency of a “small-world” brain network depends on consciousness level: a resting-state FMRI study. Cereb Cortex.

[38] Luppi AI (2019). Consciousness-specific dynamic interactions of brain integration and functional diversity. Nat Commun.

[39] Strogatz SH (2001). Exploring complex networks. Nature.

[40] Wang J (2015). GRETNA: a graph theoretical network analysis toolbox for imaging connectomics. Front Hum Neurosci.

[41] Biswal B (1995). Functional connectivity in the motor cortex of resting human brain using echo-planar MRI. Magn Reson Med.

[42] Crone JS (2014). Altered network properties of the fronto-parietal network and the thalamus in impaired consciousness. Neuroimage Clin.

[43] Northoff G, Huang Z (2017). How do the brain’s time and space mediate consciousness and its different dimensions? Temporo-spatial theory of consciousness (TTC). Neurosci Biobehav Rev.

[44] Hannawi Y (2015). Resting brain activity in disorders of consciousness: a systematic review and meta-analysis. Neurology.

[45] Wu X (2018). White matter deficits underlying the impaired consciousness level in patients with disorders of consciousness. Neurosci Bull.

[46] Di Perri C (2014). Measuring consciousness in coma and related states. World J Radiol.

[47] Fernandez-Espejo D (2011). Diffusion weighted imaging distinguishes the vegetative state from the minimally conscious state. Neuroimage.

[48] Demertzi A (2015). Intrinsic functional connectivity differentiates minimally conscious from unresponsive patients. Brain.

[49] Qin P (2021). Higher-order sensorimotor circuit of the brain's global network supports human consciousness. Neuroimage.

[50] Zhang H (2017). Posterior cingulate cross-hemispheric functional connectivity predicts the level of consciousness in traumatic brain injury. Sci Rep.

[51] Wu X (2015). Intrinsic functional connectivity patterns predict consciousness level and recovery outcome in acquired brain injury. J Neurosci.

[52] Ren X et al (2020) Robust Brain Magnetic Resonance Image Segmentation for Hydrocephalus Patients: Hard and Soft Attention. In IEEE 17th International Symposium on Biomedical Imaging (ISBI): IEEE.

[53] Mikell CB (2015). Frontal networks associated with command following after hemorrhagic stroke. Stroke.

