# Supplementary material for: Enhanced brain parcellation via abnormality inpainting for neuroimage-based consciousness evaluation of hydrocephalus patients by lumbar drainage
Source: Brain Inform. 2023 Jan 19;10(1):3. doi: 10.1186/s40708-022-00181-5 (PMC9852379; doi:10.1186/s40708-022-00181-5)
Supplement: Supplementary file 1 — Additional file 1. Clinical information of 28 patients with secondary mild hydrocephalus. [file 40708_2022_181_MOESM1_ESM.docx]

Additional file Table S1:

|  | Before LCFD | | After LCFD | |
| --- | --- | --- | --- | --- |
|  | CRS-R | Dianosis | CRS-R | Dianosis |
| Sub 1 | 23 | EMCS | 23 | EMCS |
| Sub 2 | 21 | EMCS | 23 | EMCS |
| Sub 3 | 17 | MCS+ | 20 | EMCS |
| Sub 4 | 22 | EMCS | 22 | EMCS |
| Sub 5 | 23 | EMCS | 23 | EMCS |
| Sub 6 | 8 | MCS- | 13 | MCS+ |
| Sub 7 | 5 | UWS | 5 | UWS |
| Sub 8 | 3 | UWS | 3 | UWS |
| Sub 9 | 4 | UWS | 7 | MCS- |
| Sub 10 | 22 | EMCS | 23 | EMCS |
| Sub 11 | 4 | UWS | 9 | MCS- |
| Sub 12 | 12 | MCS+ | 18 | EMCS |
| Sub 13 | 23 | EMCS | 23 | EMCS |
| Sub 14 | 8 | MCS- | 12 | MCS+ |
| Sub 15 | 20 | EMCS | 20 | EMCS |
| Sub 16 | 13 | MCS+ | 16 | MCS+ |
| Sub 17 | 9 | MCS- | 17 | MCS+ |
| Sub 18 | 7 | MCS- | 11 | MCS+ |
| Sub 19 | 19 | EMCS | 22 | EMCS |
| Sub 20 | 21 | EMCS | 21 | EMCS |
| Sub 21 | 7 | UWS | 11 | MCS+ |
| Sub 22 | 17 | MCS+ | 20 | EMCS |
| Sub 23 | 16 | MCS+ | 16 | MCS+ |
| Sub 24 | 6 | UWS | 14 | MCS- |
| Sub 25 | 5 | UWS | 5 | UWS |
| Sub 26 | 17 | MCS+ | 20 | EMCS |
| Sub 27 | 18 | MCS+ | 18 | MCS+ |
| Sub 28 | 8 | MCS- | 13 | MCS+ |
